# Physiological and Clinical Investigation of a White Dog with Blue Eyes

**Published:** 1898-08

**Authors:** 


Physiological and Clinical Investigation of a White Dog with Blue Eyes. Albinotic animals are also partly deaf and dumb. Buffon, Rawitz, Hoffmann, Fröhner, Stockfleth, Müller, Woodroffe, Hill and others have written about them. Drexler, adjunct in the Vienna Veterinary High School, owns a white dog with bright gray spots on the back. It is a male fox-terrier, deaf and dumb, but never sick, whose skin is almost wholly without pigment. The animal howls peculiarly and tries to bark, but without producing any sound. The eardrum is alive, but false. No anomalies of formation are present. The cartilaginous promontories at the base of the ear are for the most part lacking or smoothed over, the orifice into the outer ear is narrow, like a slit, the skin of the same is thin, pale, and hairless. Cerumen is not to be found. The taste and smell are normal, the eye myopic, the iris brightblue, but the reaction to light normal.—*Idem.*

Urticaria. Against itching in urticaria Dr. Berliner recommends the following treatment: The places affected should be wet with cold water and rubbed for ten to fifteen seconds with a few grains of ordinary salt taken up on the moist finger-tips. The patient feels at first a slight burning sensation, which is followed by that of a pleasant coolness and a total or very considerable decrease in the itching. At the same time the urticaria rash usually disappears rapidly. On the places that have been so treated zinc salve or rice powder should be applied. In cases of very extended urticaria it is well to proceed by zones and not treat all the places at once. If it is of poisonous origin a purgative, such as calomel, is to be recommended.—*Idem.*

